# Transcriptome analysis of *Phytolacca americana* L. in response to cadmium stress

**DOI:** 10.1371/journal.pone.0184681

**Published:** 2017-09-12

**Authors:** Yongkun Chen, Junkai Zhi, Hao Zhang, Jian Li, Qihong Zhao, Jichen Xu

**Affiliations:** National Engineering Laboratory for Tree Breeding, Beijing Forestry University, Beijing, China; INRA, FRANCE

## Abstract

*Phytolacca americana* L. (pokeweed) has metal phytoremediation potential, but little is known about its metal accumulation-related genes. In this study, the *de novo* sequencing of total RNA produced 53.15 million reads covering 10.63 gigabases of transcriptome raw data in cadmium (Cd)-treated and untreated pokeweed. Of the 97,502 assembled unigenes, 42,197 had significant matches in a public database and were annotated accordingly. An expression level comparison between the samples revealed 1515 differentially expressed genes (DEGs), 923 down- and 592 up-regulated under Cd treatment. A KEGG pathway enrichment analysis of DEGs revealed that they were involved in 72 metabolism pathways, with photosynthesis, phenylalanine metabolism, ribosome, phenylpropanoid biosynthesis, flavonoid biosynthesis and carbon fixation in photosynthetic organisms containing 24, 18, 72, 14, 7 and 15 genes, respectively. Genes related to heavy metal tolerance, absorption, transport and accumulation were also identified, including 11 expansins, 8 nicotianamine synthases, 6 aquaporins, 4 ZRT/IRT-like proteins, 3 ABC transporters and 3 metallothioneins. The gene expression results of 12 randomly selected DEGs were validated using quantitative real-time PCR, and showed different response patterns to Cd in their roots, stems and leaves. These results may be helpful in increasing our understanding of heavy metal hyperaccumulators and in future phytoremediation applications.

## Introduction

Phytoremediation is an efficient and low cost technique for removing cadmium (Cd) from contaminated soil [1–3]. Each plant species has its specific pattern, efficiency, and mechanism in Cd accumulation, transport and tolerance, especially the hyperaccumulators. Weber *et al*. [4] conducted a gene expression analysis of *Arabidopsis thaliana* and a Cd-hypertolerant variety, *Arabidopsis halleri*, under Cd treatment, and inferred that the metallophyte-specific genes might account for Cd hyper-tolerance and be indicative of toxicity mechanisms potentially involved in signaling cascades. Xu *et al*. [5] conducted a transcriptome analysis of Cd responses in *Solanum nigrum* and *Solanum torvum* with different Cd-tolerance levels. The relevant genes involved in metal transport, antioxidation, amino acid synthesis and metabolism, cell wall modification and signal transduction had higher expression levels in the Cd-tolerant ecotype. Based on a transcriptome analysis of two *Noccaea caerulescens* ecotypes, Milner *et al*. [6] identified an important root-to-shoot Cd transporter, *NcNramp1*, which had more gene copies in *N*. *caerulescens* and accounted for its function in Cd hyperaccumulation. Similar results were also reported in *Sedum alfredii* H. [7], *Populus* × *canescens* [8], *N*. *caerulescens* [9] and *Viola yedoensis* Makino [10].

*Phytolacca americana* L. (pokeweed) is a heavy metal hyperaccumulator. It can absorb large amounts of Cd and other metal ions, and transport them to shoots [11, 12]. The Cd concentration in leaves can reach 402 mg kg^–1^ when grown in heavy metal contaminated soil [13]. The majority of Cd was anchored in vacuoles in its inorganic form, or integrated into cell walls with pectates and protein [14]. Manganese (Mn), abscisic acid (ABA) and mercury (II) chloride (HgCl_2_) could significantly reduce Cd concentrations in pokeweed tissues, further improving plant growth [11, 13]. This suggested that Mn and Cd might use the same proteins for metal transport [11]. Moreover, Liu *et al*. [13] showed that ABA and HgCl_2_ could also decrease leaf transpiration and the Cd concentration in *P*. *americana* shoots, indicating that transpiration plays an important role in Cd accumulation in *P*. *americana*.

The research here explores the genes of *P*. *americana* related to Cd absorption, transport, accumulation and tolerance through a transcriptome analysis. The results contribute information on heavy metal hyperaccumulators and may lead to further phytoremediation applications.

## Materials and methods

### Plant materials and Cd treatment

Pokeweed seedlings were planted in plastic pots with 300g sandy soil (one plant per pot) in a growth chamber at normal growth temperature (25°C). Plants were irrigated daily and fertilized weekly with half-strength Hoagland’s nutrient solution [4]. In about one mouth, the identical plants with 8–10 true leaves were selected for cadmium treatment by spraying 50 mL CdCl_2_ solution (in concentration of 0, 0.3, 0.6, 0.9, or 1.2g/L) on the surface of soil per pot to the final concentration of 0, 50, 100, 150 or 200 mg/kg dry soil, respectively. Each treatment was repeated three times. The plant tissues were individually harvested in 5, 10, and 15 days for cadmium content measurement, physiological index tests, and total RNA extraction as follows.

### Measurement of cadmium content in *P*. *americana*

One-month-old seedlings were treated with CdCl_2_ solution in a final concentration of 50, 100, 150 or 200 mg/kg dried soil (described as above) for 5, 10 and 15 days, respectively. Tuberous root, fibrous root, stem and leaf samples were individually harvested and washed to remove the surface cadmium by distilled water, then dried at 105°C for 30 min at 80°C until their weights remained constant. The dry tissues were digested in HNO_3_/HClO_4_ (4:1, v/v) at 100°C for 20 min, and kept at 190°C for 60 min until the liquid evaporated. The digest was dissolved in deionized water and the Cd^2+^ content was measured using Agilent 7500 ICP-MS. The Cd content was gained based on the standard cadmium curve. The Cd transfer coefficient was calculated with the formula of Cd rate in shoots (leaves and stems) /Cd rate in roots.

### Physiological test of pokeweed respond to cadmium treatment

One-month-old seedlings were treated with CdCl_2_ solution in a final concentration of 0, 50, 100, 150 or 200 mg/kg dried soil (described as above) for 15 days, respectively. Leaves were harvested for the physiological index test as follows.

Electrolyte leakage: 20 leaf discs (0.6 cm in diameter) were collected from leaves of each treatment, immersed in 40 mL deionized water, and shaken overnight. The electrical conductivity of the solution was measured as R1. The solution containing the leaves was then boiled for 15 min, shaken overnight, and the maximum conductivity of the tissues was determined as R2. Relative electrolyte leakage (REL) was calculated using the formula REL = R1/ R2 × 100%.

Malondialdehyde (MDA) content: 0.2 g leaf tissue was homogenized in 1 mL of 10% trichloroacetic acid (TCA). After centrifugation, the supernatant was mixed with 0.6% thiobarbituric acid (TBA). The mixture was boiled for 10 min, then centrifuged. Absorbance of the supernatant at 450 nm, 532 nm, and 600 nm was measured. The MDA content was calculated as [6.45(A_532_-A_600_)-0.56A_450_] × total extract volume / total fresh sample weight.

Chlorophyll content: 0.1 g leaf tissue was immersed in 10 mL dimethylsulfoxide (DMSO) in dark for 2 days. The absorbance of the solution at 645 and 663 nm was measured. Chlorophyll a and chlorophyll b contents were calculated using the formula of Chla = (0.0127 × A663−0.00269 × A_645_) × total extract volume (mL)/total fresh sample weight (g), and Chlb = (0.0029 × A645−0.00468 × A_663_) × total extract volume (mL)/total fresh sample weight (g). The total chlorophyll content was summed as Chla + Chlb.

### RNA extraction, *de novo* sequencing and assembly

One-month-old seedlings were treated with CdCl_2_ solution in a final concentration of 0 and 50 mg/kg dried soil (described as above) for 15 days, respectively. Leaves, stems, and roots were harvested individually. Each tissue samples from 5 identical plants of each treatment were mixed for total RNA extraction using TRIzol purification system (Invitrogen, USA). All tissue RNAs from each treatment were mixed equally and named as two gene pools of PamNor and PamCd, which were further enriched by using NEBNext Poly(A) mRNA Magnetic isolation modules (NEB, E7490). RNA sequencing was conducted by Illumina HiSeqTM 2000 (Illumina Inc.). Raw reads were processed to obtain clean reads by removing low quality bases at the 3′ end and the adapter sequences. Transcriptome *de novo* assembly was carried out using Trinity software [15].

### Gene function annotation and characterization

Getorf (http://emboss.bioinformatics.nl/cgi-bin/emboss/getorf) was used to predict open reading frames (ORFs) of the unigenes. The longest ORFs of the putative genes were annotated against the non-redundant (Nr), Swiss-Prot and Gene Ontology (GO) databases using the BLAST algorithm with the typical cut-off E-value ≤ 10^−5^. The GO functional and Kyoto Encyclopedia of Genes and Genomes (KEGG) pathway enrichment analyses were performed using Blast2GO software (BioBam Bioinformatics SL).

### Differentially expressed genes (DEGs) respond to Cd in *P*. *americana*

All of the clean reads were aligned to the unigene libraries using Bowtie software [16] and the reads per kilobase per million mapped reads (RPKM) were generated to represent the expression abundance of each unigene. DEGs were identified by the false discovery rate (< 0.01 as the threshold) and RPKM difference between PamCd and PamNor (fold change ≥2).

### qRT-PCR analysis of DEGs

Total RNA of PamCd and PamNor (described as above) were reversely transcribed to cDNA using M-MLV reverse transcriptase and random primers (Promega, USA), which were further used as the qRT-PCR templates for gene expression pattern tests. qRT-PCR was performed on a Roche LightCycler 480 Real Time PCR System (Roche, Switzerland) in a final volume of 20 μl containing 2 μl of a 1/10 diluted cDNA template, 10 μl of the 2× SYBR Premix Ex Taq (Takara, Japan) and 1.5 μl (5 mM) of gene-specific forward and reverse primers (S1 Table). Three biological replication and three parallel reactions were performed. The PCR amplification followed the manufacturer’s instructions. The β-actin gene of *P*. *americana* was used as the reference.

### Statistical analysis of physiological and biochemical data

The column graphs were created by Microsoft Excel software (Microsoft, USA), multiple comparison of cadmium content and physiological and biochemical index were performed by Statistical Analysis System (SAS) (SAS, USA) PROC ANOVA, the test model was Student Newman Keuls Test (SNK) (P = 0.01, P = 0.05).

## Results

### Response to cadmium treatment of *P*. *americana* plants

Along with Cd concentration increment and the treatment time running, the Cd content in tissues of *P*. *americana* was continuously increased (Table 1), with an exception of the fibrous roots that seem to be saturated when Cd concentration up to 100mg/kg dry soil. The maximum Cd rate was 451.81mg/kg dry tissues in leaves in 200mg/kg of Cd treatment for 15 days. The fibrous roots could accumulate more Cd, averagely 2.47 folds to the other tissues of four Cd treatments in ratio. In total, the aboveground part (stem + leaf) of *P*. *americana* plant displayed high enrichment of Cd, averagely 1.28 folds to the underground part (fibrous + tuberous) of four Cd treatments in ratio. Calculation of the Cd transfer coefficient showed that the value was not identical among treatments but more than 1 mostly with the maximum of 2.25 in 200 mg/kg of Cd treatment for 15 days. Obviously, *P*. *americana* is a kind of Cd hyperaccumulator.

**Table 1:** Cadmium content and transfer coefficient in *P*. *americana*.

Concentration of CdCl_2_ (mg/kg dry soil)	Treatment time (d)	Cd content in tissues (mg/kg dry weight)				Transfer coefficient
		Fibrous root	Tuberous root	Stem	Leaf	
0	5	1.76±0.07	2.01±0.11	0.91±0.07	0.88±0.03	0.47±0.01 Aa
	10	1.85±0.06	2.03±0.14	0.91±0.07	0.91±0.12	0.47±0.03 Aa
	15	1.78±0.10	2.1±0.06	1±0.02	1.01±0.03	0.5±0.02 Aa
50	5	171.69±9.24	60.04±3.71	64.94±6.3	107.89±7.61	0.95±0.06 Bb
	10	277.99±34.64	39.48±2.96	84.19±3.34	125.32±16.58	1.56±0.19De
	15	255.13±43.01	104.17±2.76	98.38±8.24	169.76±16.47	1.13±0.14 BCbc
100	5	239.21±40.12	71.64±4.42	88.39±11.3	115.21±6.1	0.93±0.1 Bb
	10	359.82±25.81	59.22±3.39	88.67±6.65	139.52±4.31	1.22±0.05 BCcd
	15	323.84±43.28	118.34±6.78	126.93±10.07	179.22±12.64	1.05±0.08 Bbc
150	5	310.75±17.51	84.49±2.61	122.37±12.41	158±19.51	1.11±0.14 BCbc
	10	365.99±38.84	97.23±12.01	180.59±13.54	241.53±10.65	1.39±0.09 CDde
	15	276.48±32.35	151.54±7.35	216.55±16.23	285.88±36.56	1.55±0.15 De
200	5	327.63±18.87	128.29±5.09	172.89±6.1	187.11±14.85	0.99±0.07 Bbc
	10	361.32±34.76	151.82±20.08	222±4.89	259.68±28.63	1.2±0.07 BCcd
	15	330.3±36.99	175.59±21.68	329.15±31.93	461.07±14.23	2.25±0.12 Ef

Notes: All values are the mean of triplicates (±SD). Different capital letters in the column of transfer-coefficient indicate an extremely significant difference at P<0.01 while the lower-case indicate a significant difference at P<0.05 according to the SNK multiple comparison test.

**Fig 1:**
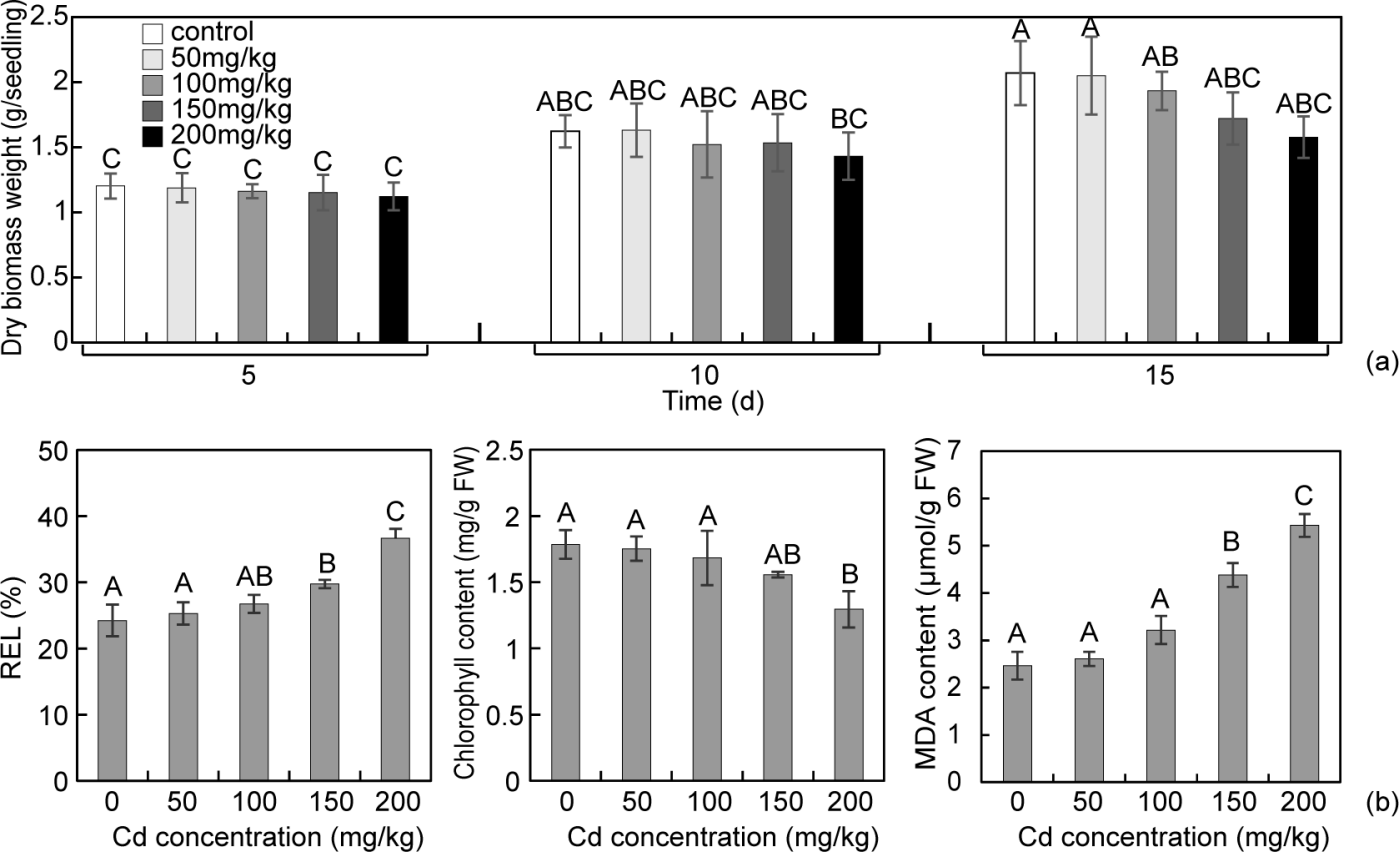
**Dry biomass production (a) and physiological indexes performance (b) of *P*. *americana* plants exposed to 0, 50, 100, 150, 200 mg CdCl**_**2**_ **/kg dry soil.** All values are the mean of triplicates (±SD). Different capital letters above the column indicate an extremely significant difference at P<0.01 according to the SNK multiple comparison test.

The dry biomass production and physiological indexes of *P*. *americana* plants in Cd treatment were tested. The dry biomass production of *P*. *americana* plants was relatively stable even no significant differences (P<0.01) was in 0–150 mg/kg dry soil Cd treatments for 10 days (Fig 1A). The data in Fig 1B clearly showed *P*. *americana* plants having strong resistance ability to Cd stress. Among three physiological indexes tested, no significant difference among 0–100 mg/kg dry soil displayed. Whereas a visible damage happened when the Cd concentration in the soil reach to 150 mg/kg, the chlorophyll content decreased 12.8% while the REL and MDA content separately increased 22.7% and 77.7%.

### *De novo* RNA sequencing and assembly of *P*. *americana*

In total, 53.15 million reads containing 10.63 gigabases of raw sequence data were obtained from both PamNor and PamCd transcriptome libraries. The ratio of Q20 (base error rate of less than 1%) reached 95%. The average double-end sequencing fragment was 100 bp in length (Table 2).

**Table 2:** Sequencing results of the *P*. *americana* transcripts with (PamCd) and without (PamNor) Cd treatment.

Sample	Total Reads	Total Bases	Mean length	GC (%)	Q20(%)
PamNor	28,284,820	5,656,226,170	100 base	46.35	95.06
PamCd	24,862,931	4,972,118,074	100 base	45.5	95.07

**Fig 2:**
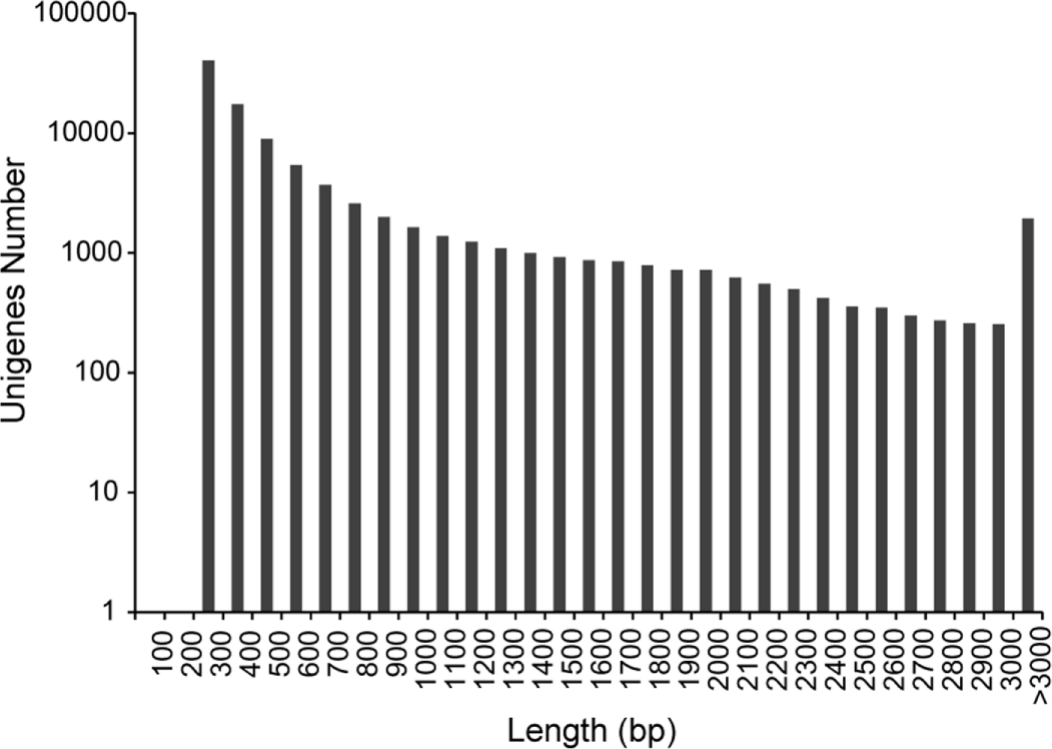
Unigene length distribution in *P*. *americana* transcripts with and without cadmium treatment.

The total reads were assembled into 134,500 transcripts accounting for 97,502 unigenes using Trinity software. On average, the N50s of transcripts and unigenes were 1605 bp and 1027 bp, respectively. For the unigenes, 31.5% were longer than 500 bp, 15.8% were longer than 1000 bp, and 2% were longer than 3000 bp (Fig 2).

Sequence alignments with NR, SWISS-PROT, GO and KEGG databases showed 42,197 annotated unigenes (43.28% of all of the unigenes). Of them, 3636 unigenes were specific to PamCd, 2673 to PamNor, and 35,888 (85%) occurred in both libraries.

### Annotation and GO analysis of DEGs in response to Cd in *P*. *americana*

**Fig 3:**
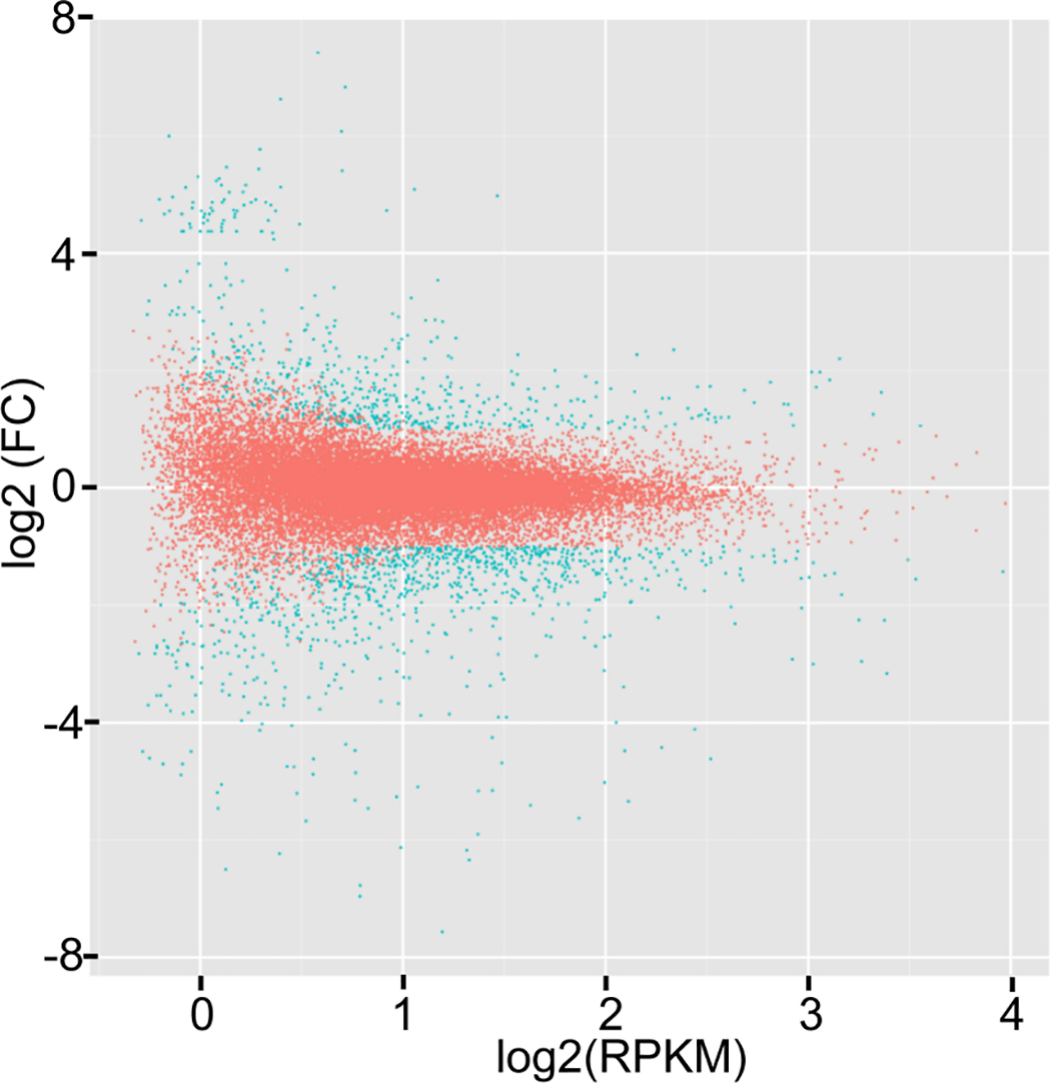
Differentially expressed genes (DEGs) between PamCd and PamNor gene pools with and without cadmium treatment. The red color represents non-DEGs, and the blue represents DEGs. The x-axis is log2 RPKM and the y-axis stand for log2 of estimated fold change.

Among the 97,502 unigenes, 1515 were detected as DEGs between PamCd and PamNor in comparison to their RPKM value. Of these, 592 were up-regulated while 923 were down-regulated under the Cd treatment (Fig 3).

Of the 1,515 DEGs, 1308 were annotated in the NR, SWISS-PROT, GO and KEGG database. Among 40 DEGs with larger expression differences, 14 genes were for ribosomal proteins, 8 for heavy metal accumulation and detoxification (such as YSL3, NAS and ABC transporters), 12 for stress tolerance (such as FLO and LRR), and genes for 3-ketoacyl-CoA synthase 5 (KCS), embryonic protein DC-8, endonuclease, ferric reduction oxidase 2, geraniol 8-hydroxylase and vegetative cell wall protein gp1.

**Fig 4:**
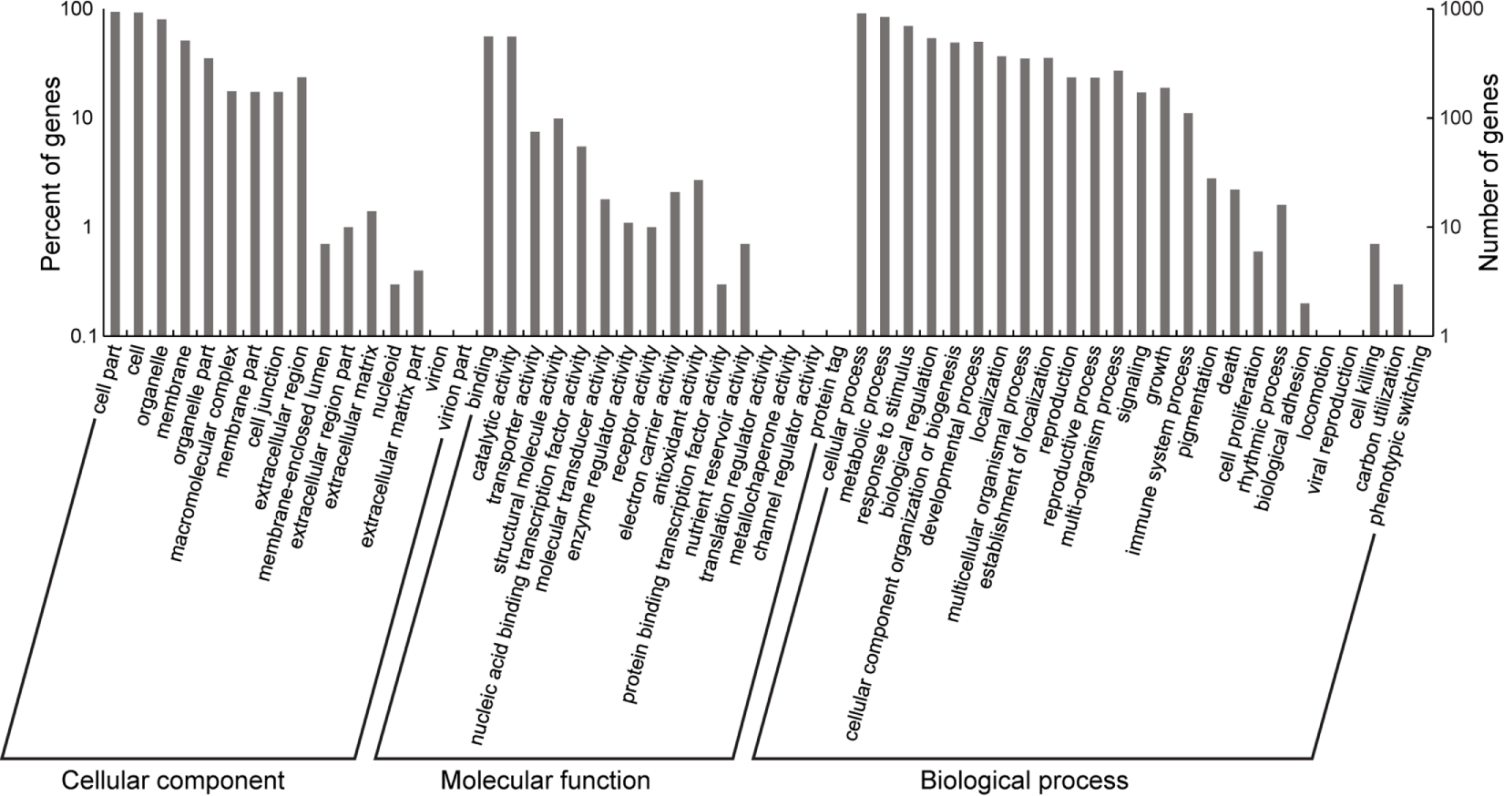
Classification of the genes in *P*. *americana* in response to cadmium via Gene Ontology (GO) analysis.

The GO functional enrichment analysis showed that 1007 DEGs were involved in biological process, 991 in cellular component, and 881 in molecular function (some DEGs overlapped among the three major categories) (Fig 4). Within the biological process, the maximum enriched gene categories included response to salt stress (110 DEGs), cold stress (99 DEGs), cadmium ion (89 DEGs), oxidation-reduction process (85 DEGs) and water deprivation (83 DEGs). Within the cellular component, the maximum enriched gene categories were in plasma membrane (229 DEGs), nucleus (185 DEGs), chloroplast (181 DEGs), plasmodesmata (173 DEGs) and membrane (155 DEGs). Within molecular function, the maximum enriched gene categories included protein binding (168 DEGs), metal ion binding (81 DEGs), structural constituent of ribosome (80 DEGs) and binding (58 DEGs).

Some DEGs were determined in relation to Cd tolerance and accumulation. Of them, 89 DEGs were involved in response to Cd ions (GO: 0046686), 81 DEGs belonged to metal ion binding (GO: 0046872) and 64 DEGs were related to various metal ion transporters. Some families of genes were down-regulated, such as nicotianamine synthase (8 members), aquaporin (7 members), Nramp (2 members) and VIT (2 members). Some families of genes were up-regulated, such as CHX (2 members) and COPT (1 member). While some families of genes showed up-regulation or down-regulation, such as ABC transporters (sub-family B members down-regulated but sub-family F members up-regulated) and expansin (A-type expansin genes down-regulated but B-type expansin genes up-regulated) (Table 3).

**Table 3:** Gene families related to heavy metal enrichment in *P*. *americana* transcriptome.

Gene Family	No. of members	Expression*	Fold Change
Expansin (EXP)	11	3 (8)	1.19–2.79
Nicotianamine synthase (NAS)	8	0 (8)	1.00–6.34
Aquaporin	7	0 (7)	1.01–2.55
Zrt/Irt-like protein (ZIP)	4	1 (3)	1.09–1.73
ATP binding cassette transporter (ABC)	3	2 (1)	2.61–6.00
Metallothionein (MT)	3	2 (1)	1.24–4.37
Aluminum-activated malate transporter (ALMT)	2	1 (1)	1.18–2.41
Cation/H(+) antiporter (CHX)	2	2 (0)	1.25–1.41
Copper transport protein (ATX)	2	1 (1)	1.06–1.09
Natural resistance-associated macrophage protein (Nramp)	2	0 (2)	1.60–1.66
Vacuolar iron transporter (VIT)	2	0 (2)	1.19–2.46
Copper transporter (COPT)	1	1 (0)	2.45
Heavy metal ATPases (HMA)	1	0 (1)	1.64
Heavy metal transport/detoxification superfamily protein (HMT)	1	1 (0)	2.35
Metal-nicotianamine transporter (YSL)	1	0 (1)	5.26

* The number outside the bracket means up-regulation while the number in bracket means down-regulation. Fold Change is the absolute value of gene expression fold change (log2) between Cd-treated and control sample.

### KEGG enrichment analysis of DEGs

A total of 20.8% of all DEGs were addressed in the 72 pathways. Of them, the ribosome pathway was enriched the most, with 72 DEGs. Amino acid synthesis and metabolism-related pathways, like phenylalanine metabolism, cysteine and methionine metabolism, arginine and proline metabolism, phenylalanine/tyrosine/tryptophan biosynthesis and tyrosine metabolism, contained 18, 11, 5, 4 and 4 DEGs, respectively. Nucleic acid-related pathways, such as RNA transport, pyrimidine metabolism and purine metabolism, contained 7, 2 and 2 DEGs, respectively. Fatty acid-related pathways, like fatty acid biosynthesis and unsaturated fatty acids biosynthesis, contained 5 and 4 DEGs, respectively. Photosynthesis-related pathways, such as photosynthesis and carbon fixation in photosynthetic organisms, contained 24 and 15 DEGs, respectively. Respiration-related pathways, like glycolysis/gluconeogenesis, oxidative phosphorylation, pentose and glucuronate interconversions, pentose phosphate pathway and glyoxylate and dicarboxylate metabolism, contained 8, 7, 6, 6 and 5 DEGs, respectively. Secondary metabolism-related pathways, such as phenylpropanoid biosynthesis, flavonoid biosynthesis, ubiquinone and other terpenoid-quinone biosynthesis, porphyrin and chlorophyll metabolism contained 14, 7, 5 and 4 DEGs, respectively. Plant hormone signal transduction-related pathways, such as plant hormone signal transduction, circadian rhythm of plant, and zeatin biosynthesis contained 10, 4, and 3 DEGs, respectively. Resistance-related pathways, like plant-pathogen interaction, glutathione metabolism, peroxisome and selenocompound metabolism, contained 6, 4, 3 and 2 DEGs, respectively.

Among all of the pathways, photosynthesis (S1 Fig) showed the most enrichment (P = 0), including 15 photosystem II genes, 3 photosystem I genes, 3 F-type ATPase genes and 3 photosynthetic electron transport genes. All of the 24 DEGs were down-regulated, which might cause the decrease in light energy absorbed and the photosynthesis efficiency when suffering from Cd toxicity. The ribosomal pathway (S2 Fig) was enriched the most, with 72 DEGs, which were associated with 46 cytoplasmic ribosomal proteins and 8 chloroplast ribosomal proteins. For the former, 36 proteins were up-regulated, while 1 was down-regulated and 9 vacillated. All eight chloroplast ribosomal proteins were down-regulated. In the ribosomal pathway, five DEGs were annotated as stress-related and three as metal ion binding-related. This indicates that the ribosomal proteins functioned not only as translation equipment, but also in plant resistance [17, 18]. Phytochelatin and metallothionein are important proteins involved in heavy metal detoxification and accumulation by chelating heavy metal ions and moving them to vacuoles for storage [19, 20]. The related pathway in *P*. *americana*, cysteine and methionine metabolism processes (S3 Fig), contained 1 up- and 10 down-regulated DEGs. All of the down-regulated DEGs were downstream of the cysteine metabolism pathway, which would aid in the accumulation of cysteine, accelerating the biosynthesis of phytochelatin and metallothionein.

### Expression testing of DEGs

**Fig 5:**
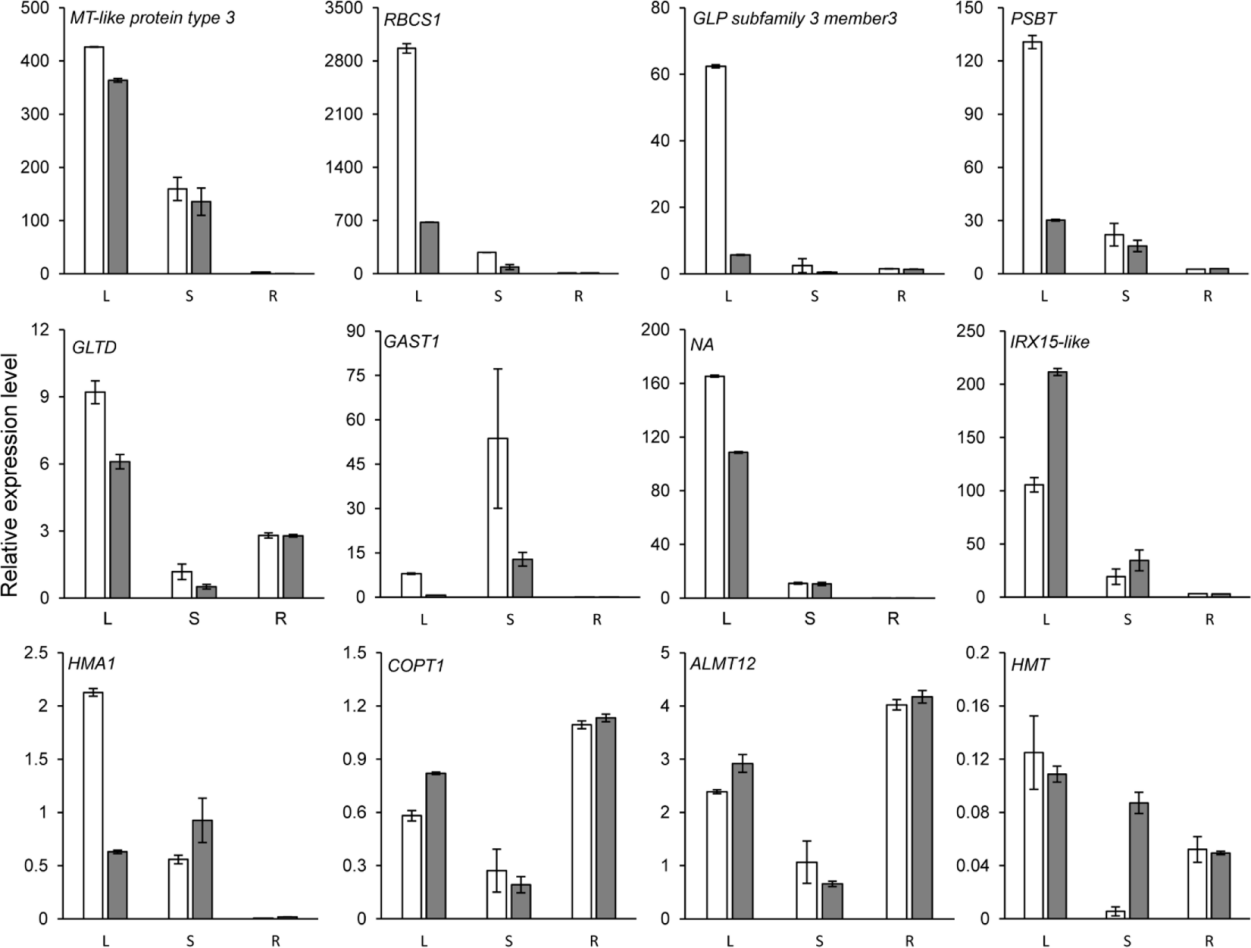
Gene expression patterns in leaf (L), stem (S), and root (R) samples of *P*. *americana* with (grey column) and without (white column) Cd treatment.

Gene expression in roots, stems and leaves of *P*. *americana* were tested using qRT-PCR (Fig 5). Results revealed the gene-specific expression patterns. Some genes, such as ribulose bisphosphate carboxylase small chain 1, showed high signals with a relative expression of 3000 compared with control leaves, while other genes, such as heavy metal transport/detoxification superfamily protein (HMT) displayed only a relative expression value of 0.15. Some genes were expressed strictly in tissues, such as metallothionein-like protein type 3 (MT3), ribulose bisphosphate carboxylase small chain 1, germin-like protein and photosystem II reaction center protein, that were highly expressed in roots but had low expression levels in stems, and gibberellic acid (GA)-stimulated transcript 1, which was highly expressed in stems compared with in other tissues. Also, different genes showed varied expression patterns, up-regulated, down-regulated, or constant under Cd treatment. Some genes were even up-regulated in one tissue but down-regulated in another tissue. Remarkably, several genes, such as HMT and Cd/zinc-transporting ATPase (HMA1), related to heavy metal ion transport were prominently up-regulated in stems, which might help Cd hyper-transport in *P*. *americana*. All of the qRT-PCR results corroborated the sequencing data.

## Discussion

A series of genes have been confirmed in relation to heavy metal absorption, root-to-shoot translocation, detoxification, and sequestration, which were slightly different in gene type, gene number, and expression ratio depending on the species. Some species such as *Phytolacca americana* [14], *Brassica juncea* [21], and *Noccaea caerulescens* [22] were considered as the hyperaccumulators which can efficiently accumulate the heavy metals in above-ground organs from soils [23] and play an important roles in phytoremediation applications. Transcriptome analysis showed that more genes in the metabolic process than in the binding process were in hyperaccumulators, including those of *V*. *yedoensis* [10] and *P*. *americana* at 1.05- and 1.31-fold, respectively, while only 0.95-fold in a non-hyperaccumulator of *Raphanus sativus* [24, 25]. The presence of these genes may help to maintain regular cell activity under heavy metal stress and facilitate heavy metal accumulation. Also, each hyperaccumulator certainly showed its own characteristics in response to heavy metal. For example, the gene numbers related to transporter activity in *P*. *americana*, *S*. *alfredii* Hance, *N*. *caerulescens* and *V*. *yedoensis* were 12.7%, 10.9%, 21.8% and 14.1% of those related to the binding process, while the gene numbers related to stimuli were 64.0%, 31.33%, 52.6% and 44.6% of those in the metabolic process[7, 9, 10]. All of above indicated that the cadmium accumulation mechanism even in hyperaccumulation was non-exclusive and complicated.

In the previous reports, some genes were analyzed for their function in heavy metal accumulation. For example, phytochelatins (PCs) can chelate heavy metal ions and/or move them into vacuoles to accumulate and reduce their toxicity [26, 27], which participated in the cysteine and methionine metabolism. Transcriptome analysis here showed a total of 11 genes involved in this process In *P*. *americana*, which could cause the accumulation of cysteine and further promote the biosynthesis of phytochelatin. This was also confirmed by a 2-dimensional gel electrophoresis (2-DE) proteome experiment [12]. Metallothionein (MT) is another chelation protein that can eliminate reactive oxygen and enhance oxidation resistance [28, 29]. The RNAi and heterologous expression experiments confirmed them could well enhances Cd tolerance and accumulation levels [30, 31]. In *P*. *americana*, 3 MT genes were involved in response to Cd treatment those belong to 3 MT families of MT1, MT2 and MT3. Of them, MT2 and MT3 were highly expressed even in the untreated sample. MT1 displayed highly inducible expression by cadmium treatment. It indicated the possible synergistic mechanism of them in Cd accumulation. Several other hyperaccumulators of *N*. *caerulescens* [9] and *Sesbania drummondii* [32] also verified the conclusion but a non-hyperaccumulator of *Medicago truncatula* disagreed [33].

The high efficiency of heavy metal transport from root-to-shoot was another prominent characteristic of hyperaccumulators. Several proteins were confirmed having this ability and well expressed in hyperaccumulator but not in non-accumulator such as heavy metal ATPases (HMA) [34, 35], natural resistance-associated macrophage proteins (NRAMP) [6], and metal-nicotianamine transporter (yellow stripe-like, YSL)[36]. In *P*. *americana*, Nramp3, Nramp6, HMA1, HMT, YSL1, YSL6 and YSL7 were highly up-regulated under cadmium treatment, especially Cd/zinc-transporting ATPase and HMT were confirmed by qRT-PCR and displayed a higher expression pattern in stems than in roots (Fig 5). Besides, 30 DEGs were also identified in divalent metal ion transport, 9 in metal ion transport, 24 in transition metal ion transport, 4 in Cd ion transport, 2 in Cd ion transmembrane transporter activity, and 2 in Cd ion transmembrane transport.

Seventeen highly expressed aquaporin genes were identified in *P*. *americana* in response to Cd treatment. The RPKM value of PIP1-5 could reach 1333, relatively 87.1-fold and 149.1-fold for the reference gamma-tubulin gene actin gene. Nevertheless, the total RPKM value of six PIP members in the non-hyperaccumulator of *M*. *truncatula* is only 46.08 in Hg-treated samples [33], and 5.76- and 7.85-fold for the reference gamma-tubulin and actin genes. Obviously, the number and expression level of PIP members are significantly correlated with the heavy metal accumulation of plants. Maurel thought that aquaporin could mediate in heavy metal ion transport and accumulation via transpiration improvement [37]. Overexpression of *Arabidopsis* plasma membrane aquaporin (PIP1b) in tobacco or tobacco aquaporin1 (NtAQP1) in tomato significantly increased transpiration rates of plants [38–40]. An aquaporin inhibitor (HgCl_2_) and ABA treatment could cause an obvious decrease of shoot Cd content along with a decrease in the transpiration rate. Some more evidences were found in Salt’s study [41], in which the ABA treatment could increase stomatal diffusion resistance and reduced transpiration rate, along with Cd accumulation in leaves was dramatically reduced. The author concluded that the Cd accumulation in hyperaccumulator of *Brassica juncea* was mainly by mass flow due to transpiration.

Our study here also revealed that a few heavy metals related genes did not show significantly different expression patterns between heavy metal hyperaccumulators and general plants in Cd treatment. Sequence alignment displayed a little variation inside. We could believe that these variations might cause an effect on the structure of the encoding proteins further lead to a change in efficiency like binding. A previous report provided convincing evidence here, in which an orthologous functional analysis of *Pid3* gene showed their resistant spectra to rice blast much differently [42]. Further exploration of these heavy metal related genes in pokeweed in base composition, codon usage bias, and protein structure would be necessary to dissect and understand the specific accumulation mechanism in hyperaccumulator.

## Conclusions

Transcriptome analysis of *P*. *americana* showed essential gene information in relation to cadmium enrichment in cadmium hyperaccumulator. The result here revealed the possible mechanism of hyperaccumulator theoretically, and provide the evidences for the molecular breeding of heavy metal phytoremediation.

## Supporting information

S1 Fig The photosynthesis pathway responding to Cd treatment in *P*. *americana*. (TIF)

S2 Fig The pathway of ribosome metabolism responding to Cd treatment in *P*. *americana*. (TIF)

S3 Fig The pathway of cysteine and methionine metabolism responding to Cd treatment in *P*. *americana*. (TIF)

S1 Table The qRT-PCR primers for candidate DEGs and reference *Actin* gene. (PDF)
