# Correction: Transcriptome analysis of *Phytolacca americana* L. in response to cadmium stress

**DOI:** 10.1371/journal.pone.0199721

**Published:** 2018-06-21

**Authors:** Yongkun Chen, Junkai Zhi, Hao Zhang, Jian Li, Qihong Zhao, Jichen Xu

The Data Availability statement for this paper is incorrect. The correct statement is: Sequencing data are available within NCBI BioProject under accession PRJNA449977. All other relevant data are within the paper and its Supporting Information files.
