# Supplementary figures and images for: Transcriptome analysis of Phytolacca americana L. in response to cadmium stress
Source: PLoS One. 2017 Sep 12;12(9):e0184681. doi: 10.1371/journal.pone.0184681 (PMC5595333; doi:10.1371/journal.pone.0184681)

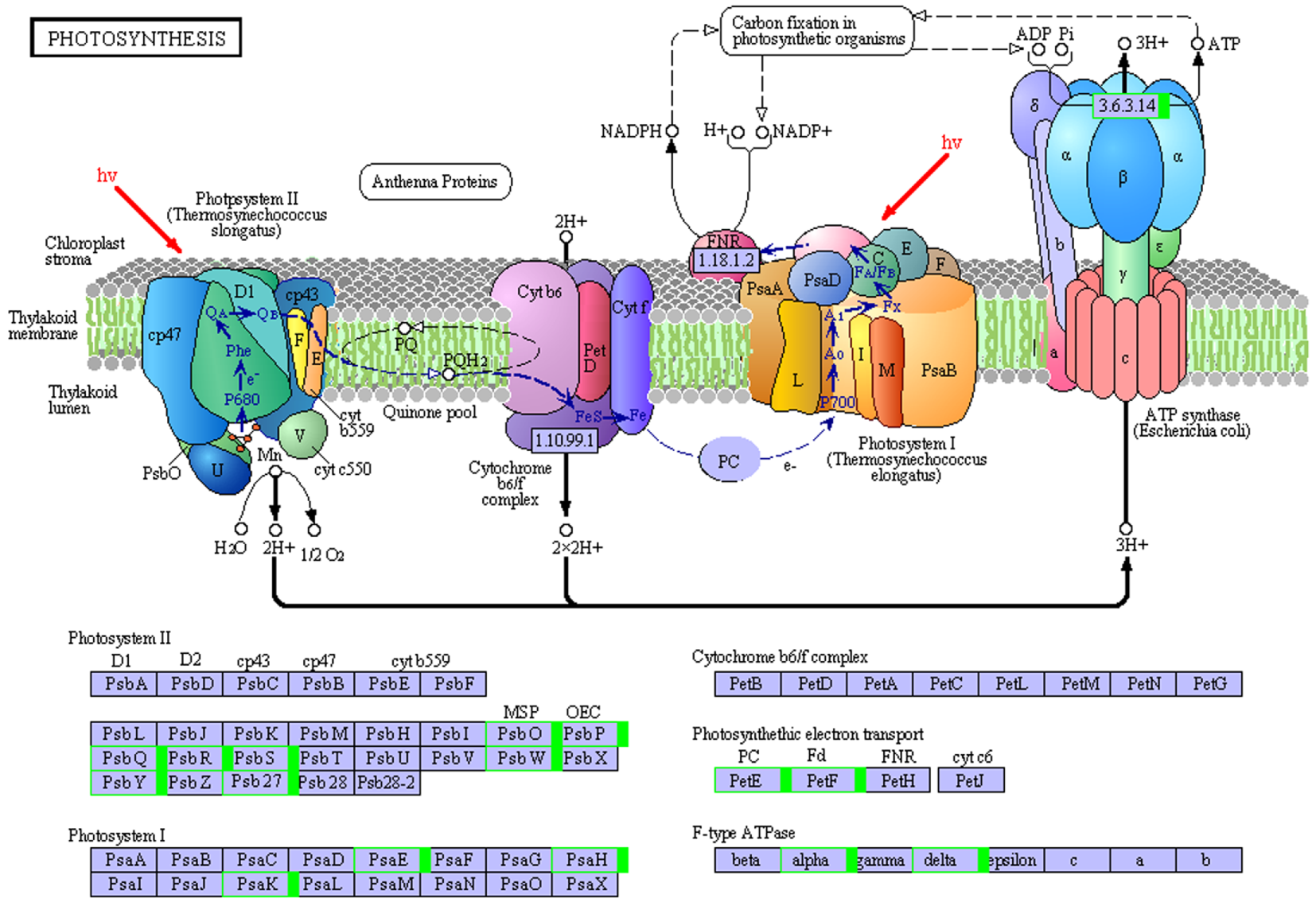

Supplement: S1 Fig — (TIF) [file pone.0184681.s001.tif]

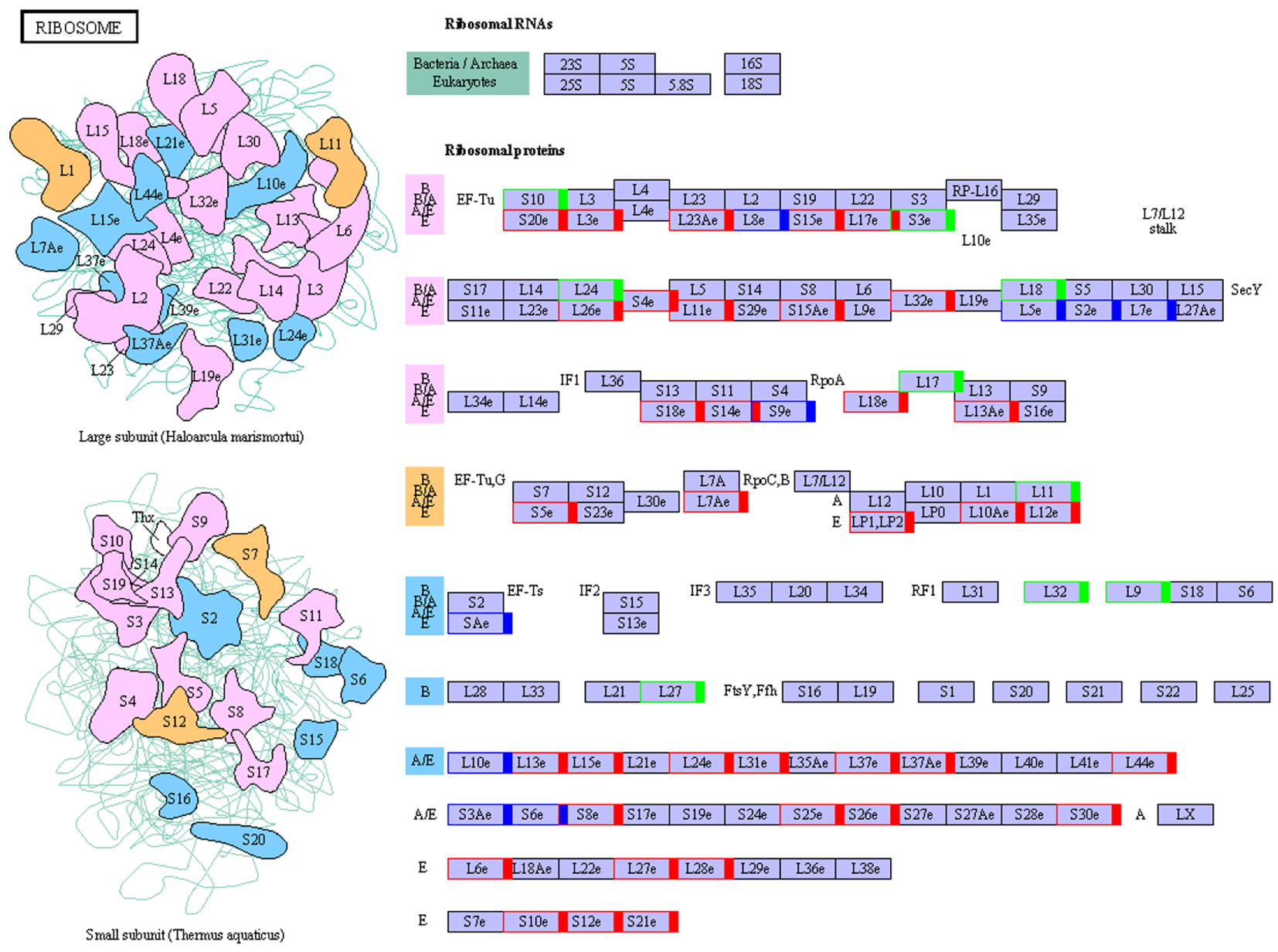

Supplement: S2 Fig — (TIF) [file pone.0184681.s002.tif]

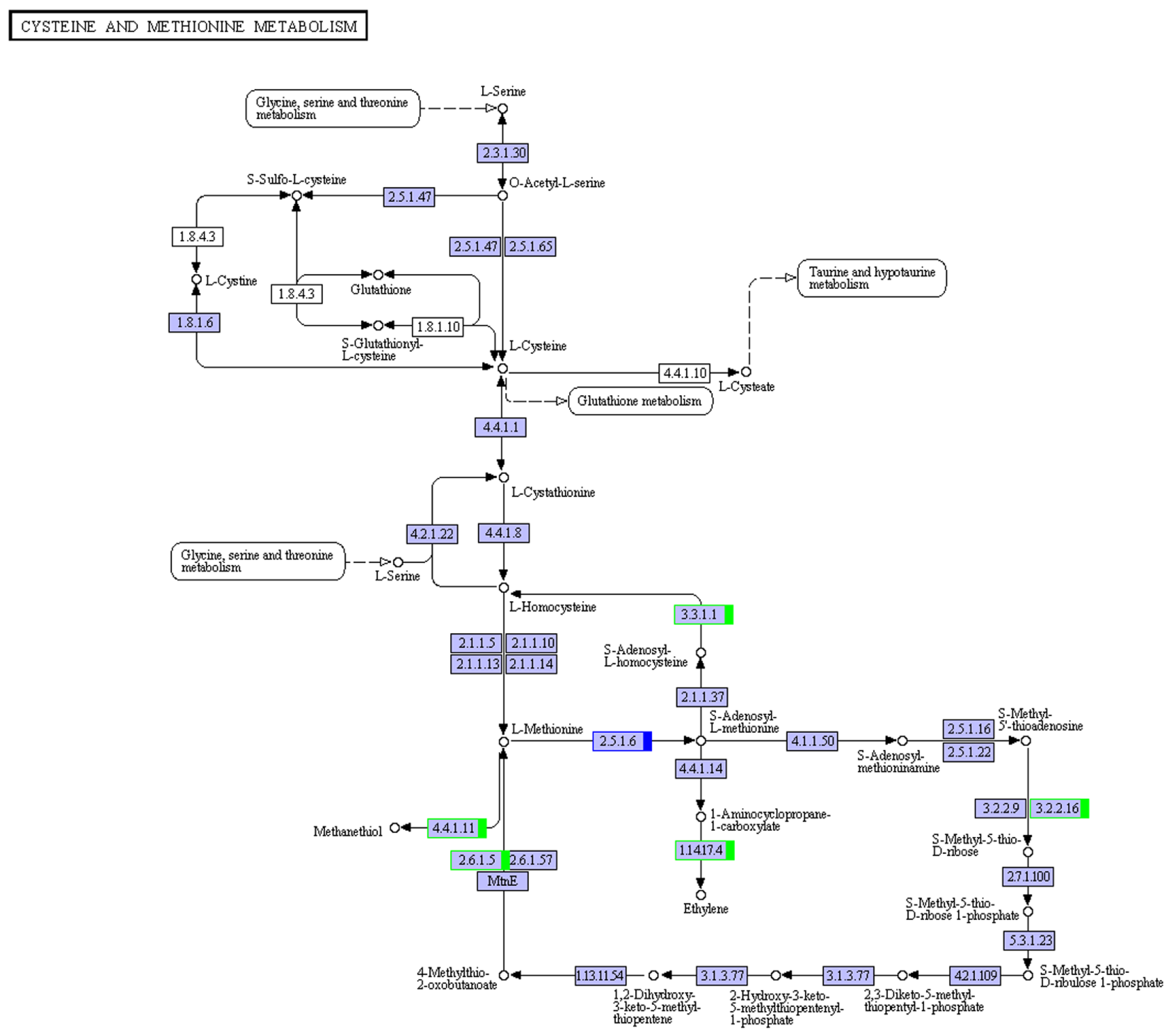

Supplement: S3 Fig — (TIF) [file pone.0184681.s003.tif]
