# Supplementary material for: Transcriptome analysis of Phytolacca americana L. in response to cadmium stress
Source: PLoS One. 2017 Sep 12;12(9):e0184681. doi: 10.1371/journal.pone.0184681 (PMC5595333; doi:10.1371/journal.pone.0184681)
Supplement: S1 Table — (PDF) [file pone.0184681.s004.pdf]

S1 Table. The qRT-PCR primers for candidate DEGs and reference Actin gene.

Gene	Forward primer	Reverse primer
GAST1	AGTCAATCTGCTCAGCCACAAGG	CTGCTTGTTACCATAGGTGCCAG
RBCS1	CGGGCTTGTAGGCAATGAAACTG	CACGGATTTGTGTACCGTGAGAAC
MT like 3	GCTCCTCAATCTTACTTGCATCCC	GTGACATCCACATCCGCAGTTAG
PSBT	CATCCGTGTCTCAGAGAAACAGTC	TGGCAACACAAACAGGTGAATAGG
HMA1	GCACATGGTGATGCTGCCACCAT	CATGTCCTCATGGCTCTTGCAGC
GLP	TACACGCCATTGGCTGATGATATG	GCTGCTTGTTACCATAGGTGCCAGG
GLTD	TACCCTCGATCGATTTCGATCC	CCGAGCTTCATGATTGCCTTCTC
NA	CTGGGTCTTTGCATGGGATCATAG	GAAGGTATTGGATCGTGGCCTTG
COPT1	TGTCATCGTCGAGTGGCTATCA	GAGTCCCAAACACAAGGAACCC
ALMT12	TTGAGACCGACATTGAGCAAGA	TTCAACGGGTCGCTCATTACA
HMT	CCACAGTAAGAATCTGAGCACC	CATTCATTGTGACAAGGACAGGC
IRX15	TGGCATCAGTGCAAGTACAAGTAG	GCATTGGTGTCCGATGTGGGTA
